# Using Persuasive Systems Design Model to Evaluate “Cuida tu Ánimo”: An Internet-Based Pilot Program for Prevention and Early Intervention of Adolescent Depression

**DOI:** 10.1089/tmj.2018.0272

**Published:** 2020-02-05

**Authors:** Fernando Parada, Vania Martínez, H. Daniel Espinosa, Stephanie Bauer, Markus Moessner

**Affiliations:** ^1^ Doctoral Program in Psychotherapy, Faculty of Medicine and Faculty of Social Sciences, Universidad de Chile and Pontificia Universidad Católica de Chile, Santiago, Chile.; ^2^ Millennium Nucleus to Improve the Mental Health of Adolescents and Youths, Imhay, Santiago, Chile.; ^3^ Millennium Institute for Depression and Personality Research (MIDAP), Santiago, Chile.; ^4^ Faculty of Medicine, Centro de Medicina Reproductiva y Desarrollo Integral del Adolescente (CEMERA), Universidad de Chile, Santiago, Chile.; ^5^ Faculty of Psychology, Universidad CES, Medellín, Colombia.; ^6^ Center for Psychotherapy Research, University Hospital Heidelberg, Heidelberg, Germany.

**Keywords:** adolescence, depression, e-mental health, internet, telemedicine, telehealth

## Abstract

***Background:*** “Cuida tu Ánimo” (CTA [Take Care of Your Mood]) is an internet-based program for prevention and early intervention of adolescent depression implemented in Chile and Colombia. In the pilot application of the program, participants interacted with the program through a website that provided psychoeducational information, chat, and telephone appointments as well as monitoring and feedback messages. To date, most similar programs were not developed taking design features into consideration. The persuasive systems design (PSD) model is a comprehensive framework developed to aid in the design and evaluation of systems capable of influencing users' attitudes or behaviors. The purpose of this study was to evaluate the persuasiveness of CTA pilot program using the PSD model.

***Methods:*** The methodology used was expert evaluation, where specialists evaluate the program against a list of design principles.

***Results:*** Although the PSD model was not used to design the program, system features proposed by PSD were present, mainly “Dialogue support” features. Persuasion context analysis was not carried out by the developers. No aspects of the program could be related to “Primary task support” features because the developers did not define a primary task.

***Discussion:*** Key aspects of the PSD model could be incorporated in the CTA program to enhance system persuasiveness and improve adherence.

## Introduction

### Adolescent Depression

Adolescent depression is a worldwide issue.^1^ It has been associated with significant general impairment, greater risk of smoking, substance misuse, and obesity, and more than half of adolescent suicide victims reported to have a depressive disorder at the time of death.^1–4^ Despite this fact, adolescent depression is missed more often than in adults, possibly because of its similarity to adolescence itself and also because of the lower percentage of adolescents seeking help to cope with symptoms.^1^ Furthermore, access to proper treatment is limited in many countries, making research in novel strategies to detect and intervene adolescent depression a matter of significant importance.^1^ Electronically and online-delivered interventions and assessment methods, also called e-mental health (eMH), are gaining importance as evidence grows and shows promising results for diverse populations, including adolescent depression.^5,6^

### Internet-Based Interventions (eMH)

eMH interventions are being increasingly studied globally, mainly because of their advantages in terms of access, cost-effectiveness, and ubiquity.^6,7^ These strategies can be used to deliver information, monitoring, screening, evaluation, intervention, and social support.^8,9^ For adolescents, several eMH clinical trials have been conducted with promising results for anxiety and depression (e.g., Online Cognitive Behavioral Therapy Programs have shown to be effective in reducing anxiety and depressive symptomatology).^5^

### “Cuida Tu ánimo” Program

“Cuida tu ánimo” (CTA [Take Care of Your Mood]) is an internet-based program for the prevention and early intervention of adolescent depression implemented in Chile and Colombia (www.cuidatuanimo.org). The study was approved by the Human Research Ethics Committee of the Faculty of Medicine, Universidad de Chile, Chile, and Institutional Committee on Human Research Ethics Universidad CES, Colombia. Adolescents from four schools were invited to the study. They interacted with the program via monitoring and feedback messages, which were delivered every 2 weeks, and a website that allowed them to access psychoeducational content. In addition, a section of the website provided emergency information and allowed users to contact a specialist via online chat appointments. Adolescents with severe depressive symptoms or suicidal risk were invited to participate in an online chat appointment, a phone session, or a face-to-face assessment with a mental health professional, as required by each case. To promote use, several visits were made to schools that participated in the study, in which staff members distributed bracelets and handouts with information about the program along with other merchandising products.

One of the main issues found in the study was adherence. Adherence to eMH interventions is defined by their intended usage, which refers to the extent to which the intervention should be used in order for participants to get maximum benefit from it.^10^ Low adherence indicators were observed in the CTA program, with an average of 2 monitoring e-mails answered by adolescents, with a maximum of 16, and an average of 4 clicks made in the website, with a maximum of 55, which mean significantly low website exploration and usage.

Poor adherence is a common problem of eMH strategies and needs to be addressed. A review by Kelders et al.^10^ revealed that the adherence rates of internet interventions reached a maximum of 50% of intended usage. Also, it was observed that intervention characteristics and persuasive technology elements could predict adherence.^8^

### Persuasive Systems Design

Persuasive systems design (PSD) is a holistic framework for devising persuasive technology.^11^ The system design features were proposed by Oinas-Kukkonen and Harjumaa^11^ and implemented by Adaji and Vassileva.^12^

This model classifies the features of persuasive technology in four main categories.

### PSD Features

- Primary task support: Design principles that support the carrying out of a primary task by the user.
- Dialogue support: Principles related to the implementation of human-computer dialogue in a way that helps users move toward their goal or target behavior.
- System credibility support: Principles that describe how to design a credible system, which will be more likely to persuade its users.
- Social support: Principles that describe how to design a system so that it motivates its users by leveraging social influence.

A brief description of the above-mentioned design principles can be found in *Table 1*.

**Table 1.** System Design Features Proposed by the Persuasive Systems Design Model

PRIMARY TASK SUPPORT	DIALOGUE SUPPORT	SOCIAL SUPPORT	SYSTEM CREDIBILITY SUPPORT
Reduction: From complex to simple, accomplishable tasks.	Praise: Using compliments with users to augment persuasion.	Social learning: Means to observe other users performing target behavior and to see the outcomes of their behavior are desirable.	Trustworthiness: Providing information that is truthful, fair, and unbiased.
Tailoring: Fitting the user's needs as an element of persuasion.	Rewards: Providing rewards for target behavior.	Social comparison: Comparing own performance with other users'.	Expertise: Providing information showing knowledge, experience, and competence.
Tunneling: Guiding users through the process.	Reminders: Reminding users of elements related to the primary task.	Normative Influence: Gathering together people who have the same goal and make them feel norms.	Surface credibility: Competent look and feel.
Personalization: Offering the possibility of personalized content.	Suggestion: Suggestions could elicit target behavior more effectively.	Social facilitation: Identifying other users who are performing the behavior.	Real-world feel: Delivering information about the organization and/or people behind its contents and services.
Self-monitoring: Allowing users to monitor their progress on their own.	Similarity: Systems that remind users of themselves in a meaningful way are more likely to persuade.	Cooperation: Providing means for cooperation.	Authority: Referring to people in the position of authority.
Simulation: Providing simulations to enable users to observe the link between cause and effect immediately.	Liking: Having an appealing look and feel for users.	Competition: Providing means for competing with other users.	Third-party endorsement: Presenting endorsements from respected sources.
Rehearsal: Providing means to practice target behavior.	Social role: System should adopt a social role.	Recognition: Providing public recognition for users who perform their target behavior.	Verifiability: Users should be able to verify the accuracy of site contents via outside sources.

Adapted from Oinas-Kukkonen and Harjumaa.^11^

Persuasive technology elements along with intervention characteristics could account for a substantial amount of variance in adherence rates to eMH interventions.^10^ Thus, applying this model to internet-based interventions could be helpful to see how system design features are used and how they influence adherence. The objective of this study was to evaluate the persuasiveness of the CTA program by applying the PSD model as an evaluation framework,^12^ with the aim to produce insights and suggestions on how to improve the program's design features to promote adherence.

## Methods

Expert evaluation was conducted by the authors, F.P. and V.M., to compare and cross-check design features included in the CTA program with those proposed by the original PSD article.^11^ The program was evaluated in terms of its system design features.^11,12^

## Results

Several design principles were found in the CTA program, such as Rewards, Reminders, Suggestions, Similarity, Liking, Trustworthiness, Expertise, Real-World Feel, and Third Party Endorsements, all of which are included in the “Dialogue support” and “System credibility support” categories. Nevertheless, only two design principles, Self-Monitoring and Social Learning, of the “Primary task” and “Social support” categories were identified. This indicates that CTA provided a pleasant visual environment for the adolescents to receive the intervention, as well as means to complete assessments and interact with the website content, but was more ineffective at proposing specific easy-to-accomplish tasks for its users and did not allow participants to interact with each other in any way. *Table 2* shows the design principles found in CTA and the aspects of the program related to them.

**Table 2.** Persuasive Systems Design Features Found in “Cuida tu Ánimo” Program

PRIMARY TASK SUPPORT	DIALOGUE SUPPORT	SOCIAL SUPPORT	SYSTEM CREDIBILITY SUPPORT
Self-monitoring: The program allows participants to answer an online questionnaire to assess their mood, which e-mails them a report with their results.	Rewards: Bracelets, posters, stickers, and other merchandising products were handed to participants to introduce and promote participation in the program.	Social learning: Some of the educational videos on the website show adolescents making healthy decisions.	Trustworthiness: The program only presents evidence-based and unbiased information.
	Reminders: Frequent e-mail reminders were sent to participants to promote self-monitoring and exploration of the website.		Expertise: The information in the program is provided by mental health professionals.
	Suggestion: The program provides suggestions for preventing mood changes and coping with aversive experiences.		Real-world feel: Website has a section about the CTA research team and its affiliations.
	Similarity: The characters in the program's videos and images are the same age as the adolescent population with mood disorders and have similar issues.		Third-party endorsement: Website shows endorsement by Universities, a research group, and government agencies.
	Liking: The website is colorful and was designed to appeal to adolescents.		

CTA, “Cuida tu ánimo.”

## Discussion

PSD principles were not taken into account when CTA was designed. Nevertheless, several features of the PSD model were found, which indicated that CTA provided adequate feedback to its users and contained information from reliable and professional sources, which could have made it more likely to persuade its users by supporting its credibility. In addition, CTA was designed in a colorful and appealing manner, rewarded its users for participating in the program and provided reminders and suggestions for them to navigate the site adequately.

However, other important features were missing. The program was not effective in proposing a task for the users to perform, and it did not promote interaction between users since it was mostly informative, providing fewer opportunities to deploy social skills such as competition or cooperation.

Suggestions derived from these results could be the inclusion of more personalized content, in order for users to interact with a more familiar interface; adding concrete tasks for users to perform during their interaction with the program (e.g., enabling interactive questionnaires to test users knowledge after they watch the educational videos), providing a supervised platform for group chat, where adolescents do not only interact with therapists exclusively but also with each other, providing a sense of familiarity and closeness to the program; and rewarding users for the completion of tasks with virtual or real-life prizes (e.g., giving users points for each video watched, allowing them to exchange such points for new colors or background images for their user interface). These examples of Primary task and Social Support features could help improve the CTA program in terms of its persuasiveness with little structural changes in the website, making users more likely to adhere to the program and perform the tasks proposed by the developers.

PSD could be useful for the evaluation of internet-based interventions, providing structured and concise information on system features and how to improve interventions in terms of their persuasiveness.
